# Assessing the Joint Value of Genomic-Based Diagnostic Tests and Gene Therapies

**DOI:** 10.3390/jpm9020028

**Published:** 2019-05-21

**Authors:** Sean P. Gavan, Christine Y. Lu, Katherine Payne

**Affiliations:** 1 Manchester Centre for Health Economics, Faculty of Biology, Medicine and Health, The University of Manchester, Oxford Road, Manchester M13 9PL, UK; katherine.payne@manchester.ac.uk; 2 Department of Population Medicine, Harvard Medical School and Harvard Pilgrim Health Care Institute, Boston, MA 02215, USA; christine_lu@harvardpilgrim.org

**Keywords:** diagnostic test, gene therapy, health economics, opportunity cost, value assessment frameworks

## Abstract

Gene therapy is an emerging type of treatment that may aim to provide a cure to individuals with a genetic mutation known to be causative of a specific disease. A diagnosis of the causative mutation must precede treatment with a in vivo gene therapy. Both achieving a genomic-based diagnosis and treatment with a gene therapy may result in substantial expenditures for health care systems. Uncertainties around the health care costs, risks, and benefits derived from diagnosis and treatment with a subsequent gene therapy suggests a need for developing an evidence base, underpinned by opportunity cost, to inform if, and how, these health technologies should be introduced into health care systems funded by finite budgets. This article discusses why current methods to evaluate health technologies (decision-analytic model-based cost-effectiveness analysis from the perspective of a health care system over a lifetime time horizon) are appropriate to quantify the costs and consequences of using genomic-based diagnostic tests and gene therapies in combination, rather than as separate interventions, within clinical practice. Evaluating the economic impact of test-and-treatment strategies will ensure that the opportunity cost of these health technologies is quantified fully for decision-makers who are responsible for allocating limited resources in health care systems.

## 1. Introduction

Gene therapy is an emerging type of treatment that aims to provide a definitive cure to individuals with a genetic mutation known to be causative of a specific disease. The regulatory definition of a gene therapy is a medicine that includes “an active substance which contains or consists of a recombinant nucleic acid used in or administered to human beings with a view to regulating, repairing, replacing, adding or deleting a genetic sequence” [1]. Gene therapies often use a vector, which is generally a viral delivery system, to administer treatment. The premise of curative intent indicates that gene therapies could be administered as a one-time treatment during a person’s lifetime [2].

The safety profile of gene therapies has started to improve due to changes in the viral vectors used to insert genes [3]. Regulatory processes are in place to mitigate the risk to patients and to ensure the safe use of gene therapies. In Europe, gene therapies are categorized under the regulatory remit of a broader classification called “advanced therapy medicinal products” (ATMPs) [4]. The relevant regulatory office in the USA is the Center for Biologics Evaluation and Research (CBER) within the Food and Drug Administration [5]. A relatively high number of gene therapies, however, have failed to reach the market because of the toxicity associated with the viral vector or due to poor commercial viability. In 2012, the gene therapy Glybera achieved marketing authorization to treat familial lipoprotein lipase deficiency (LPLD). Glybera was marketed at a price of €1.1 million per patient [6]. In October 2017, the manufacturer, uniQure, did not pursue marketing re-authorization due to a lack of demand for the treatment [7]. A gene therapy in-development may also reach a patient population on an experimental or “compassionate use” basis if no authorized treatment alternatives are available and the patient population is not able to enter a clinical trial [8,9]. 

Danzon & Towse highlighted the potential economic value of gene therapies in 2002 [10]; but only more recently have practical examples of gene therapies become available for clinical application (see Table 1). Initial gene therapies developed in the 1980s were based on ex vivo approaches to treat conditions such as severe combined immunodeficiency [2]. Newer developments have focused on in vivo approaches, which involve the direct insertion of genes that function correctly aimed at rare inherited diseases or “anticancer” genes. Although the number of licensed gene therapies is still relatively small, there is an active agenda for research to develop hundreds of new potential gene therapies in the public and private sectors [11].

A definitive diagnosis, informed by a genomic-based testing strategy, that provides information about the specific causative genetic mutation must precede treatment in order to identify the population of patients who will benefit from a in vivo gene therapy. For example, a group of inherited eye conditions classified under “retinitis pigmentosa” cause a progressive loss of vision resulting in bilateral blindness [12]. Over 3000 genetic mutations have been identified as being responsible for retinitis pigmentosa [12]. Genetic heterogeneity introduces the challenges of, firstly, being able to identify the defective gene to replace or silence and, secondly, to use the correct treatment strategy for the causative gene. Strategies to identify causative genetic mutations have improved following the development of next-generation sequencing (NGS) technologies [13]. These NGS technologies have stimulated the clinical application of genomic-based diagnostic tests [14]. Gene panel tests are now available to detect the mutation in the genes that cause retinitis pigmentosa [15] to enable subsequent treatment with gene therapies such as Luxturna.

A genomic-based diagnostic test that directs patients to a relevant gene therapy is similar to the use of the term “companion diagnostic”. Companion diagnostics are tests that are essential for the safe and effective use of a treatment. Companion diagnostics may be co-developed with their respective treatment or developed independently following evidence that patients with specific characteristics may have improved response to treatment or fewer adverse events [16]. The term “companion diagnostic” has not yet been used in the context of gene therapy [17]. Genomic-based diagnostic tests enter clinical practice when there is evidence that a specific gene may be responsible for a defined phenotype (the genetic condition) and are not developed with the intent of being companion diagnostics. Genomic-based diagnostic tests are developed predominantly as laboratory (or in-house) tests [14,18] and their regulatory process focuses on quality assurance and quality control processes of the laboratory [19]. Frameworks to evaluate genomic-based diagnostic tests, such as the “analytic validity, clinical validity, clinical utility, and ethical, legal, and social implications” (ACCE) framework proposed by the United States Centres for Disease Control and Prevention, underpin existing processes to inform the introduction of new tests into health care systems [20,21]. However, these frameworks currently do not consider evidence of the opportunity cost imposed by a new genomic-based diagnostic test. 

## 2. Opportunity Cost and Value Assessment Frameworks

The cost of gene therapies, genomic-based diagnostic tests using NGS, and gene panel tests represent substantial expenditures for health care systems [14,22]. Within this context, decision-makers who are responsible for recommending health technologies in public and private health care systems must consider appropriate strategies for funding, coverage, and reimbursement of genomic-based diagnostic tests and any associated gene therapies that are consistent with the objectives of the health care system (for example, the maximization of health) [23]. Mechanisms to absorb the high cost of gene therapies have been suggested, such as using a capped annuity with a risk-sharing agreement (continued annual payment whilst the treatment effect is maintained), but these mechanisms have been viewed with skepticism [2]. 

Budgets for health care are finite; in turn, the resources required to provide any new health technology will be obtained from the resources used to provide health care elsewhere in the system. In making coverage and reimbursement decisions with a budget-constrained health care system, decision-makers must consider the benefits forgone due to displacing a health technology in order to allocate those same resources towards a new health technology (“opportunity cost”) [24]. Opportunity cost within the context of allocating resources for health care is usually expressed in terms of the health benefits forgone [25]. Decision-makers can maximize benefits from health care at the population-level by using economic evidence to understand whether the (health) benefit derived from a new health technology is greater than its opportunity cost [26]. Methods of economic evaluation, such as decision-analytic model-based cost-effectiveness analysis, provide a mechanism for generating this economic evidence [24]. 

Uncertainties around the health care costs, risks, and benefits derived from achieving a genetic diagnosis and receiving treatment with a subsequent gene therapy suggests a need for developing an evidence base, underpinned by opportunity cost, to inform if, and how, these health technologies should be introduced into health care systems funded by finite budgets. The opportunity cost of gene therapies has the potential to be substantial due to their high price range that will subsequently require other patients in the health care system to forgo treatments. Explicit value assessment frameworks have been proposed to aid decision-makers responsible for allocating limited resources for health care in determining whether specific treatment strategies represent good value to the health care system [27].

A number of distinct value assessment frameworks are now in use within and across countries and health care jurisdictions [28,29]. Disease-specific value assessment frameworks have been developed to help decision-makers assess the value of treatments for people with a specific disease. These disease-specific value assessment frameworks comprise a suite of potential benefits deemed to be of value. For example, the American Society of Clinical Oncology Value Framework assesses therapies for cancer with respect to their magnitude of clinical benefit, toxicity, and cost [30]. Similarly, the European Society for Medical Oncology Magnitude of Clinical Benefit Scale assesses therapies for cancer with respect to their clinical benefit, toxicity, and impact on quality of life [31]. 

Generic value assessment frameworks, which can be used across a range of diseases, have also been developed to assess the value of specific types of health technology. These frameworks use evidence from decision-analytic model-based cost-effectiveness analyses to estimate the incremental costs and health consequences of a new health technology relative to an appropriate comparator. For example, the National Institute for Health and Care Excellence (NICE) in England has assessment and appraisal programs for technologies, in general, and for diagnostics, specifically. These value assessment frameworks are underpinned in theory by health opportunity cost and require evidence of the relative impact on health gain, measured using quality-adjusted life years (QALYs), given the relative cost to the health care system [32,33]. A threshold approach is used by the NICE appraisal process, set within the range of £20,000 to £30,000 per QALY gained, to establish whether the technology or diagnostic is a cost-effective use of resources [26]. NICE has also developed discrete value assessment frameworks for specific types of technologies such as the NICE Highly Specialized Technologies (HST) program which can evaluate medicines with a high unit cost for very rare (ultra-orphan) conditions [34]. The NICE HST program uses a different threshold for relative cost-effectiveness (£100,000 per QALY gained) and also has different criteria for assessment including the nature and extent of treatment alternatives, the magnitude of QALY gain, and the extent of disease morbidity and clinical disability [34]. In the USA, the Institute for Clinical and Economic Review (ICER) is an independent non-profit organization that has also produced value assessment frameworks [35]. The core ICER value assessment framework evaluates the clinical and economic value of prescription drugs, medical tests, and other health technologies using the criteria of short-term affordability (budget impact analysis), long-term value (comparative effectiveness and incremental cost-effectiveness), as well as other wider benefits (for example, a reduction in the burden for caregivers) and contextual factors (for example, the first treatment to offer any improvement for patients or whether there is a high lifetime burden of illness). The threshold for cost-effectiveness used by ICER falls within the range of $50,000 to $150,000 per QALY gained [35]. ICER has also defined a value assessment framework for very rare diseases that uses a wider threshold range of $50,000 to $500,000 per QALY gained whilst accounting for additional contextual factors (the impact on patient and caregiver productivity, education, disability, and nursing home costs) [36]. Paulden et al. [37] reported a detailed scoping review of the value arguments (for example, magnitude of benefit, severity of disease, availability of treatment alternatives) that have been proposed elsewhere in the literature with respect to the reimbursement of orphan drugs.

The current trend of disease-specific and technology-specific value assessment frameworks may present difficulties to decision-makers who are responsible for allocating resources to health technologies across a range of diseases. For example, decision-makers will need to make implicit trade-offs between different outcomes across diseases if disease-specific value assessment frameworks comprise different attributes of benefit. Technology-specific thresholds for cost-effectiveness, such as those for orphan drugs, will also be detrimental to population health if they are set too high because the health forgone due to the displacement of health technologies will exceed the health gained from the new health technology [38].

## 3. Assessing the Value of Genomic-based Diagnostic Tests and Gene Therapies

**Figure 1:**
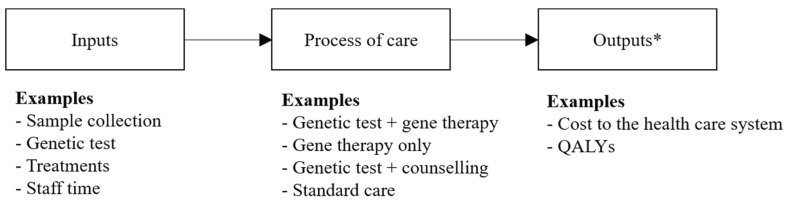
Link between Health Care Inputs, the Process of Care, and Outputs Relevant to Decision-makers. * Outputs are consistent with the perspective of the analysis (e.g., a health care system perspective), measured over the duration of the time horizon (e.g., a lifetime time horizon), and discounted to their present value.

Previous commentators have suggested appropriately that existing value assessment frameworks, such as the ones used by NICE, are fit for the purpose of evaluating the costs and consequences of gene therapies [39]. There are, however, some important nuances that need consideration in the application of existing value assessment frameworks within the context of genomic-based diagnostic tests and gene therapies. The use of gene therapy, in practice, can be described as a complex intervention with at least two independent components; the diagnosis and the therapy (see Figure 1) [40]. 

Complex interventions can include interventions with more than one interacting component such as a test-and-treatment strategy that provides information to inform prescribing behavior. Costs and health consequences can accrue from each component when used in combination or sequentially within a pathway of care [41]. Decision-makers require end-to-end evidence over the complete diagnosis and treatment pathway in order to inform resource allocation decisions within a health care system [42]. The NICE Diagnostics Assessment Programme is a good example of how end-to-end evidence is used to inform decision making; assessments can consider the economic impact of using different testing strategies (sequence of testing, timing of testing, different diagnostics) and different treatment or management strategies [32]. 

In the context of gene therapy, gene panel tests that test multiple genes simultaneously can be used to achieve a genetic diagnosis. Multiple tests of single genes, by contrast, can be time-consuming, relatively expensive, and have variable uptake across the health care system [42]. Costs to the health care system and health consequences will be derived from both the testing strategy and the gene therapy component when combined in a model of service delivery. The current approach of evaluating genomic-based diagnostic tests and gene therapies independently is therefore inadequate for decision-makers because it does not fully account for the impact of using these health technologies in combination on costs and health consequences. 

Zimmerman et al. [43] have published a decision-analytic model-based cost-effectiveness analysis of a in vivo gene therapy (voretigene neparvovec) for people with RPE65-mediated inherited retinal disease in the USA health care system. The incremental costs and QALYs of voretigene neparvovec, compared with standard of care, were $825,621 and 1.3 QALYs respectively. However, the target population was defined as already having a diagnosis of biallelic RPE65-mediated inherited retinal disease. In practice, a genetic testing strategy is necessary to identify this target population but this was omitted from the economic evaluation. The diseased population may have been a more appropriate target population for the study to account for the use of the genetic diagnostic test and the impact of the test results on a subsequent decision to prescribe a gene therapy. Therefore, the analysis may have underestimated the resources required to deliver gene therapy as a complex intervention within the USA health care system and may have had an impact on QALYs gained if inaccuracies were present in the testing strategy to identify the relevant population with the appropriate variant.

A distinct value assessment framework is not necessary to quantify the joint value of genomic-based diagnostic testing and treatment with a subsequent gene therapy. Existing jurisdiction-specific value assessment frameworks can be used to evaluate the full pathway of care, from an initial genetic diagnosis to a treatment decision, by considering genomic-based diagnostic tests and gene therapies as an inextricably linked combination of health technologies. Figure 1 illustrates the link between the required inputs, process of care, and the subsequent costs and health consequences of achieving a genetic diagnosis and receiving treatment with a gene therapy within the framework of a cost-effectiveness analysis. The relative cost-effectiveness of the combined diagnostic and treatment components will be affected by factors such as the prevalence of a particular genetic mutation, the unit and analysis costs associated with the testing strategy, the clinical accuracy and predictive ability of the diagnostic test, the capacity to analyze samples in a timely manner within routine clinical practice, the availability of a gene therapy and its respective cost, effectiveness, and duration of benefit. 

The choice of genomic-based diagnostic test and the clinical evidence supportive of gene therapies, however, introduce some practical challenges when estimating the cost-effectiveness of care pathways that include gene therapies. Only patients with the specific genetic diagnosis in a particular population (true-positives) will benefit from a gene therapy following a genomic-based diagnostic test. Patients who do not have this genetic diagnosis after testing (true-negatives) will be ineligible for the specific gene therapy that targets a particular mutation. Inaccuracies in the testing strategy may lead some patients (false-positives) to receive the gene therapy inappropriately or some patients (false-negatives) to not receive the gene therapy when they would have otherwise benefited from treatment. The value of the health technology for those “ineligible” patients may be linked to subsequent genetic counselling rather than the relative effectiveness of treatment with a gene therapy [14]. In the absence of treatment, outcomes that extend beyond the domain of health may be proposed to enter the value assessment framework, such as a reduced time to diagnosis or the ability for patients to make informed life decisions [14]. Decision-makers may, in turn, require evidence of the health-related benefits forgone due to allocating resources to strategies that improve consequences not related to changes in health [41]. Similarly, if such consequences are measured as a benefit within a value assessment framework, they should also be considered as potential benefits forgone within the estimation of opportunity cost [44]. 

A value-assessment framework used to estimate the relative cost-effectiveness of a genomic-based diagnostic test and gene therapy, when compared with current practice, should quantify uncertainty explicitly. Estimates for the relative effectiveness of gene therapies are characterized by substantial uncertainty at product launch [45,46]. Rare inherited conditions, often the target population for gene therapies, are characterized by a small number of patients within a health care system that may limit the feasibility of recruiting a sample to a randomized controlled trial. Randomization to gene therapy or placebo may be deemed “unethical” if no other therapeutic alternative is available or if patients have severe disease [47]. Instead, single-armed trials that compare the outcomes post-gene therapy with the outcomes observed during an earlier time period are often used to produce evidence of effectiveness [45]. The effectiveness of gene therapy is frequently estimated using surrogate end-points because the long-term outcomes purported by curative gene therapies may not be observable within the duration of a trial [47]. Therefore, decision-makers will require explicit evidence that changes in a surrogate end-point lead to the improvement of an outcome that is important for reimbursement (for example, life expectancy or QALYs) [47]. Economic analyses can also be performed iteratively to quantify the need to collect additional data due to the impact of these uncertainties in the clinical evidence base on the likelihood that a gene therapy is cost-effective [48,49]. 

The evaluation of genomic-based diagnostic tests and gene therapies in combination is likely to be the responsibility of the decision-makers who make recommendations about the allocation of resources for health care systems. A genomic-based diagnostic test and gene therapy may be manufactured by different entities such as a pharmaceutical manufacturer with a laboratory-developed test or distinct pharmaceutical and diagnostic companies. However, this separation of the manufacturing process does not preclude the assessment of these health technologies when used in combination by a national decision-maker. Different testing strategies and management strategies should be compared using a fully incremental cost-effectiveness analysis. Furthermore, recommendations made by decision-makers can be reappraised over time as new gene therapies (for a particular variant) or genomic-based diagnostic tests (to detect a particular variant) enter the market.

## 4. Conclusions

The current discussions about the need to produce evidence to understand the economic impact of introducing expensive gene therapies have focused on the cost-effectiveness of the therapy component in isolation. Gene therapies can only be effective if the appropriate target population has been identified correctly; this relies on achieving an accurate diagnosis informed by the use of a genomic-based testing strategy. Methods to assess the value of gene therapies must, therefore, be capable of quantifying the economic impact of the combined diagnostic and treatment elements. This test-and-treatment approach does not accord with the emergence of technology-specific value assessment frameworks and technology-specific thresholds for “cost-effectiveness”. Evaluating the diagnostic and gene therapy in combination will ensure that the opportunity cost of these health technologies, expressed in terms of health forgone, is quantified fully for decision-makers who are responsible for allocating finite budgets within health care systems. 

## Figures and Tables

**Table 1:** Examples of Licenced Gene Therapies.

Condition	Therapy	Administration	Status	Price Per Dose
Vision loss	Luxturna (voretigene neparvovec-rzyl)	In vivo	Licensed 2017	USD$850,000
Lipoprotein lipase deficiency	Glybera (alipogene tiparvovec)	In vivo	Licensed 2012; withdrawn 2017	€1.1million
Severe combined immunodeficiency	Strimvelis (autologous CD34+)	Ex vivo	Licensed 2016	€594,000
